# Single Molecule Imaging Reveals Differences in Microtubule Track Selection Between Kinesin Motors

**DOI:** 10.1371/journal.pbio.1000216

**Published:** 2009-10-13

**Authors:** Dawen Cai, Dyke P. McEwen, Jeffery R. Martens, Edgar Meyhofer, Kristen J. Verhey

**Affiliations:** 1 Department of Cell and Developmental Biology, University of Michigan, Ann Arbor, Michigan, United States of America; 2 Department of Pharmacology, University of Michigan, Ann Arbor, Michigan, United States of America; 3 Department of Mechanical Engineering, University of Michigan, Ann Arbor, Michigan, United States of America; 4 Biophysics Research Division, University of Michigan, Ann Arbor, Michigan, United States of America; Adolf-Butenandt-Institut, Germany

## Abstract

Cells generate diverse microtubule populations by polymerization of a common α/β-tubulin building block. How microtubule associated proteins translate microtubule heterogeneity into specific cellular functions is not clear. We evaluated the ability of kinesin motors involved in vesicle transport to read microtubule heterogeneity by using single molecule imaging in live cells. We show that individual Kinesin-1 motors move preferentially on a subset of microtubules in COS cells, identified as the stable microtubules marked by post-translational modifications. In contrast, individual Kinesin-2 (KIF17) and Kinesin-3 (KIF1A) motors do not select subsets of microtubules. Surprisingly, KIF17 and KIF1A motors that overtake the plus ends of growing microtubules do not fall off but rather track with the growing tip. Selection of microtubule tracks restricts Kinesin-1 transport of VSVG vesicles to stable microtubules in COS cells whereas KIF17 transport of Kv1.5 vesicles is not restricted to specific microtubules in HL-1 myocytes. These results indicate that kinesin families can be distinguished by their ability to recognize microtubule heterogeneity. Furthermore, this property enables kinesin motors to segregate membrane trafficking events between stable and dynamic microtubule populations.

## Abstract 2

Molecular motors differentially recognize and move cargo along discrete microtubule subpopulations in cells, resulting in preferential transport and targeting of subcellular cargoes.

## Author Summary

Eukaryotic cells assemble a variety of cytoskeletal structures from a set of highly conserved building blocks. For example, all microtubules are generated by the polymerization of a common α/β-tubulin subunit, yet cells can contain diverse, discrete populations of microtubule structures such as axonemes, spindles, and radial arrays. This diversity must be read and translated by cellular components in order to carry out population-specific functions. We use single-molecule imaging to study how molecular motors navigate the heterogeneous microtubule populations present in interphase cells. We show that different kinesin motors select different subpopulations of microtubules for transport. This selectivity, based solely on the motor-microtubule interface, may enable kinesin motors to segregate transport events to distinct microtubule populations and thus to target cargoes to specific subcellular destinations.

## Introduction

Understanding how cells generate intracellular structures and overall morphologies is one of the major goals of cell biology. For the cytoskeleton, strikingly different structures can be assembled from a set of highly conserved building blocks. For example, all microtubules are generated by the polymerization of a common α/β-tubulin subunit yet diverse microtubule populations can be generated (e.g., axonemes, spindles, and radial arrays) that carry out distinct functions.

One way microtubule diversity can be characterized is based on dynamic properties [1]. Some microtubules are dynamic and turn over rapidly by alternating between periods of microtubule growth (polymerization) and shrinkage (depolymerization). Other microtubules are stable (low turnover) and are defined by their resistance to drugs that result in depolymerization of microtubules, such as nocodazole. In vivo, microtubules frequently pause, undergoing neither polymerization nor depolymerization [2]. Microtubule diversity can also be characterized by structural differences, for example alterations in protofilament number, as well as by chemical differences between tubulin subunits due to differences in the expression of tubulin genes (isotypes) or the presence of post-translational modifications (PTMs) [1],[3]–[5].

What are the biological functions of microtubules diversity? Dynamic instability allows microtubules to explore three-dimensional space for rapid remodeling of the cytoskeleton during processes such as spindle assembly and cell migration [6]–[9]. Stable microtubules likely play important roles in cellular morphogenesis but how and why remain unclear [10]–[13]. The chemically diverse PTMs that mark stable microtubules may affect morphogenesis by stabilizing microtubules and/or by influencing distinct intracellular transport events [4]–[6]. The expression of tubulin isotypes can influence polymerization dynamics and plays a role in the formation of specific microtubule assemblies such as the flagellar axoneme [3],[14].

The challenge is to explain how the diversity of microtubule structures is translated into specific cellular functions. This likely requires the functions of a large number of microtubule associated proteins (MAPs). Of special interest are motor proteins of the kinesin and dynein families that use ATP hydrolysis to move cellular cargoes along microtubule tracks [15],[16]. The molecular and mechanistic properties of motor proteins have typically been studied in vitro using homogeneous microtubule assemblies. Thus, understanding how motor proteins could read microtubule diversity has been difficult to answer. We recently developed techniques for tracking kinesin motors at the single molecule level in the cytoplasm of live cells [17]. Here, we extend these techniques to two-color tracking and evaluate the motility of kinesin motors along heterogeneous populations of microtubules tracks in COS cells. We show that Kinesin-1 motors move preferentially along stable microtubules marked by PTMs whereas Kinesin-2 (KIF17) and Kinesin-3 (KIF1A) motors can utilize dynamic microtubule tracks. These results indicate that kinesin motors have evolved to recognize specific microtubule subpopulations and, thus, segregate membrane trafficking events within cells.

## Results

### Single Molecule Imaging of Kinesin-1 in Live COS Cells

**Figure 1:**
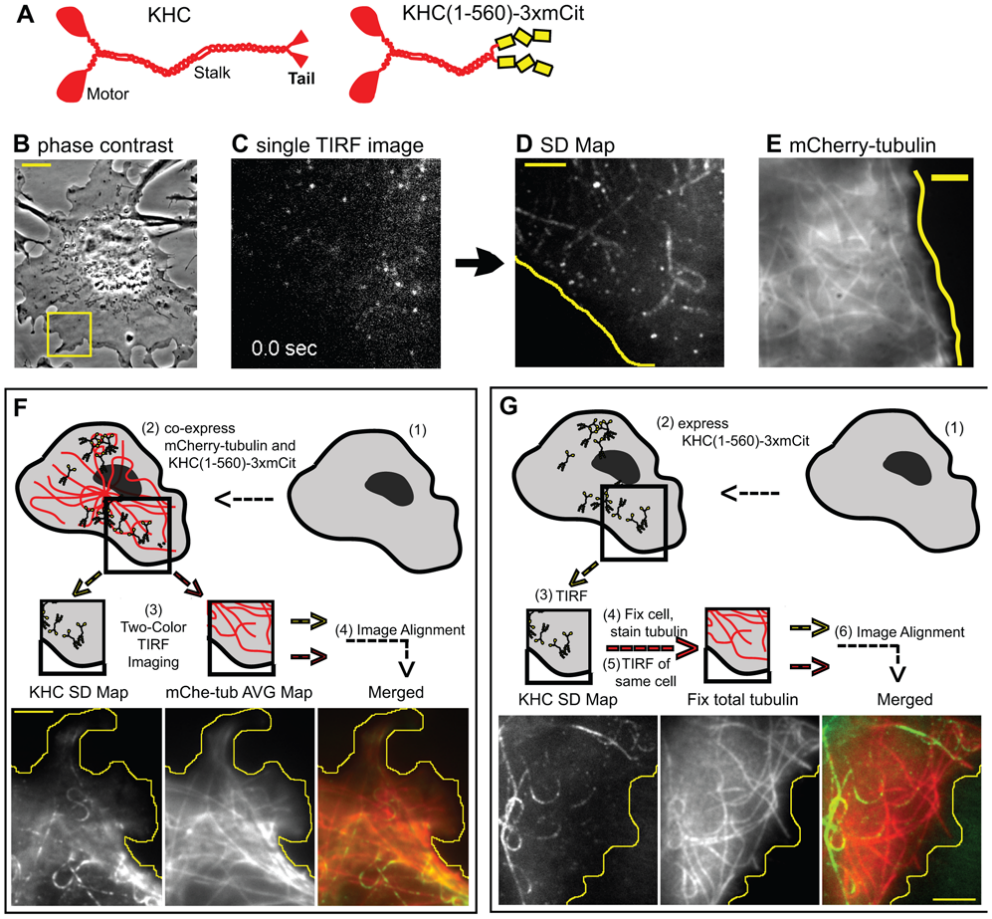
Single molecule imaging reveals preferential motility of Kinesin-1 on a subset of microtubules. (A) Schematic illustration of the domain structure of the KHC subunit of Kinesin-1 (left) and the constitutively active, truncated version KHC(1-560)-3xmCit (right). (B) Phase contrast image of a COS cell expressing KHC(1-560)-3xmCit. Scale bar, 10 µm. TIRF microscopy (C) was performed on a small region in the periphery of the cell (e.g., boxed area in B). From the image series, a SD Map (D) was created that provides a visual readout of all of the Kinesin-1 motility events. Scale bar, 3 µm. (E) TIRF image of a COS cell expressing mCherry-tubulin. Scale bar, 3 µm. (F) Two-color TIRF imaging. (1) COS cells coexpressing (2) KHC(1-560)-3xmCit and mCherry-tubulin were (3) imaged in the TIRF microscope. A SD Map of KHC(1-560)-3xmCit motility (KHC SD Map) and an average map of mCherry-tubulin (mChe-tub AVG Map) were created. The two images were (4) aligned and merged (Merged). Scale bar, 4 µm. (G) Retrospective immunofluorescence. (1) COS cells expressing (2) KHC(1-560)-3xmCit were (3) imaged by TIRF microscopy. The cells were (4) fixed immediately, stained with antibodies to total tubulin, and then (5) the same cell was imaged again in the TIRF microscope. A SD Map of the KHC(1-560)-3xmCit motility events was (6) aligned with the total tubulin image to create a merged image (Merged). Scale bar, 4 µm. Yellow lines in (D–G) indicate the edge of the cell.

Constitutively active kinesin motors can be generated by truncations that remove autoinhibitory and cargo-binding regions of the polypeptide. For this work, we generated KHC(1-560) (Figure 1A), a dimeric motor that has been well characterized in vitro and in vivo [16],[18],[19]. KHC(1-560) motors were tagged with three tandem copies of monomeric Citrine (mCit), a variant of enhanced yellow fluorescent protein (FP) (Figure 1A), and expressed in COS cells (Figure 1B). Single Kinesin-1 motors were tracked in live cells using a modified TIRF microscope (Figure 1C) in which the angle of illumination was varied to enable deeper imaging as described [17]. KHC(1-560)-3xmCit motors were observed to undergo both free diffusion and linear movement (Video S1). Linear motility occurred with an average speed of 0.83±0.08 µm/sec and average run length of 0.91±0.23 µm in live cells (Table 1, *n* = 372 events), consistent with previous work [16],[17].

**Table 1:** Summary of the motile properties of kinesin motors in live cells.

	Average Speed (µm/sec)	Average Processivity (µm/run)	% Overlap with mCherry-Tubulin	% Overlap with EB3-mRFP	% Overlap with Acetylated Tubulin
Kinesin-1	0.83±0.08	0.91±0.23	9.1±8.1	2.5±1.1	90.3±5.5
Kinesin-2	1.31±0.05	0.56±0.22	98.1±3.6	94.5±5.4	92.1±5.8
Kinesin-3	1.82±0.04	0.55±0.19	100	96.1±3.8	100

COS cells expressing Kinesin-1 [KHC(1-560)-3xmCit], Kinesin-2 [KIF17(1-490)-3xmCit], or Kinesin-3 [KIF1A(1-393)-3xmCit] motors were imaged by single molecule TIRF microscopy. From the image series, the average speed and processivity (run length) of the motors was determined. The percentage overlap with the indicated microtubule populations in live (mCherry-tubulin and EB3-mCherry) and fixed (acetylated tubulin) cells was calculated from the ratio of the (number of microtubules with motility events) to (number of microtubules).

To gain an understanding of the ensemble characteristics of Kinesin-1 motility events, we developed methods to sum all motility events of a time series into one image called the standard deviation map (SD Map) [17]. We compute this map by determining the standard deviation (SD) of the fluorescence intensity change for each pixel over the entire time series (Figure S1). Thus, pixels with little to no fluorescence variation over the time series have low SDs whereas pixels with large fluctuations in intensity (e.g., where motility events occurred) are highlighted in the SD Map. As seen in the SD Map of a single molecule TIRF movie of KHC(1-560)-3xmCit in live COS cells (Figure 1D), multiple individual Kinesin-1 motors moved repeatedly on linear tracks. Only a few Kinesin-1 tracks were identified in each image series suggesting that the SD Map reveals “hot spots” of Kinesin-1 motility. That these hot spots are not due to TIRF imaging of microtubules at the bottom of the cell is indicated by the observation of many microtubules in a comparable area of COS cytoplasm (Figure 1E). We hypothesized that Kinesin-1 motors move preferentially on only a subset of microtubule tracks.

### Kinesin-1 Motors Move Preferentially on a Subset of Microtubule Tracks

To directly test the possibility that Kinesin-1 motors distinguish microtubule populations in cells, we used two approaches. We first performed two-color TIRF imaging (Figure 1F) of live cells expressing FP-tagged Kinesin-1 motors and microtubules. This approach allows us to simultaneously visualize motors and their tracks and can account for shifts in the position of individual microtubules during live cell imaging (see [20] and Figure S2). COS cells were first transfected with plasmids encoding mCherry-tubulin and 24 h later with plasmids encoding KHC(1-560)-3xmCit. After an additional 5 h of expression, the cells were imaged by TIRF microscopy. KHC(1-560)-3xmCit motility events were observed along mCherry-tubulin microtubules (Video S2). Merged images of the SD Map of KHC(1-560)-3xmCit motility events and the mCherry-tubulin fluorescence (Figure 1F, representative of *n* = 4 cells in two experiments) indicate that mCherry-tubulin microtubules overlapped with 96.6%±5.2% of the Kinesin-1 tracks. In contrast, Kinesin-1 motility events were observed on only 9.1%±8.1% of the mCherry-tubulin microtubules (Table 1). These data indicate that Kinesin-1 motors utilize only a subset of the available microtubule tracks.

We then performed retrospective immunofluorescence (Figure 1G) after single molecule TIRF imaging. This approach avoids difficulties with double transfection and the possibility that mCherry-tubulin does not incorporate into all microtubules yet is hindered by the fact that cells shrink during fixation, often resulting in a shift in position of the entire microtubule population. For retrospective imaging, COS cells expressing KHC(1-560)-3xmCit motors were imaged by TIRF microscopy, fixed, and then stained with an antibody to total tubulin (Figure 1G). The previously imaged cell was again observed by TIRF microscopy. A comparison of the SD Map of Kinesin-1 motility events and the total tubulin staining confirmed that the motility events occurred on only a subset of microtubules present in the imaging field (Figure 1G, representative of *n* = 16 cells in six experiments). Taken together, these results confirm that Kinesin-1 motors preferentially utilize only a subset of the microtubules present in COS cells.

### Kinesin-1 Motors Do Not Move Preferentially on Dynamic Microtubules

**Figure 2:**
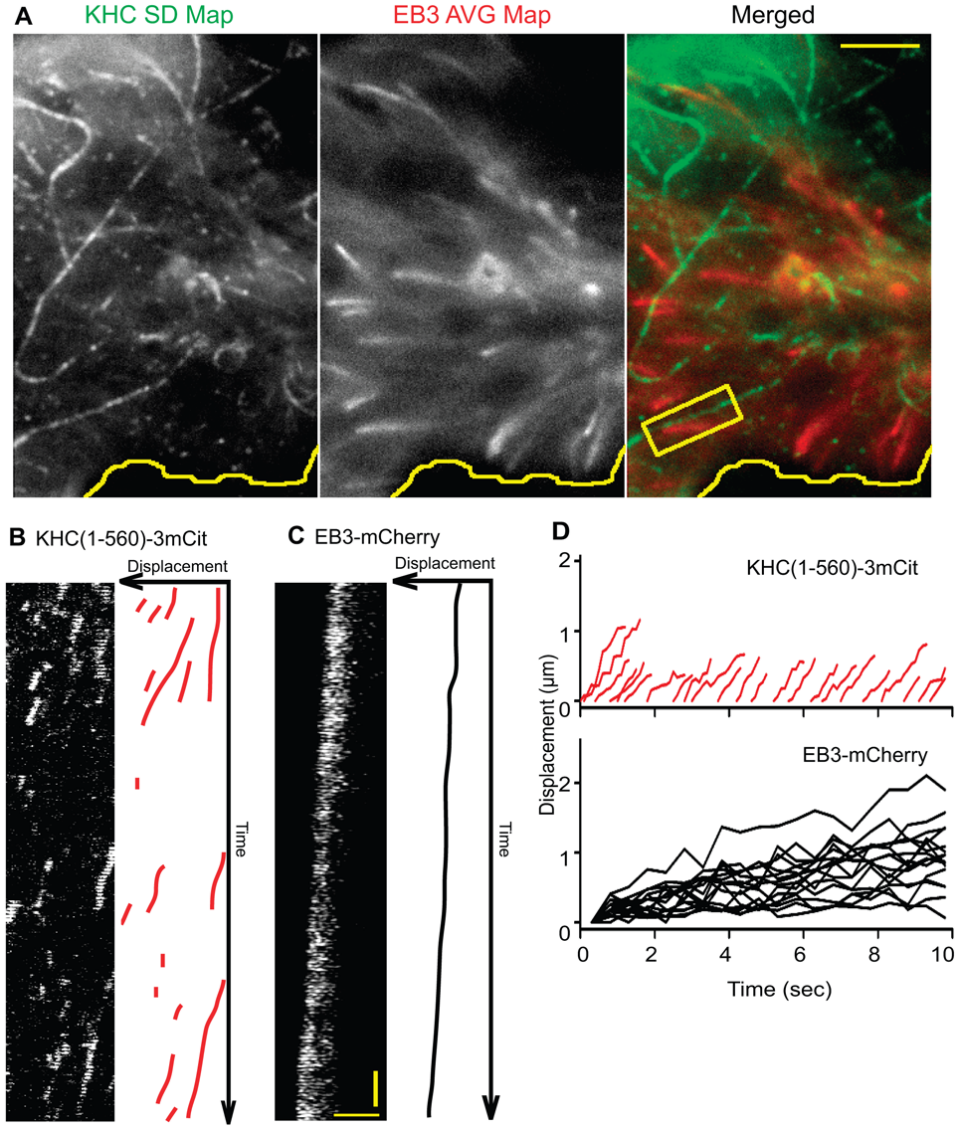
Preferential motility of Kinesin-1 does not occur on dynamic microtubules marked by EB3. (A) Two-color TIRF imaging of a COS cell coexpressing KHC(1-560)-3xmCit and EB3-mCherry. After imaging, a SD Map of KHC(1-560)-3xmCit motility events (KHC SD Map) and an average map of the EB3-mCherry fluorescence (EB3 AVG Map) were created and merged (Merged). Yellow line, edge of cell. Scale bar, 4 µm. In the boxed region, multiple KHC(1-560)-3xmCit motility events can be observed to occur adjacent to an EB3-mCherry-marked microtubule. (B) The KHC(1-560)-3xmCit motility events in the boxed region of (A) are depicted in the kymograph (left) and schematic representation (right). (C) Movement of EB3-mCherry in the boxed region of (A) is depicted in the kymograph (left) and schematic representation (right). Vertical scale bar, 0.5 s. Horizontal scale bar, 2 µm. (D) Schematic summary of the displacement over time of multiple KHC(1-560)-3xmCit motors and EB3-mCherry plus-ends in the cell in (A).

We first tested whether Kinesin-1 motors move preferentially on dynamic microtubules. This population can be observed in live cells expressing FP-tagged plus-end tracking proteins (+TIPs) [21]. Two-color TIRF microscopy was used to analyze COS cells coexpressing KHC(1-560)-3xmCit with a mCherry-labeled version of the +TIP protein end binding (EB)3 [22]. Very few Kinesin-1 motility events could be observed on microtubules extending back from the EB3-mCherry-labeled plus ends (Video S3). A comparison of the SD Map of KHC(1-560)-3xmCit motility events with the average EB3-mCherry fluorescence (Figure 2A, representative of *n* = 12 cells in four experiments) demonstrates that the microtubule tracks utilized by KHC(1-560)-3xmCit motors are distinct from the dynamic microtubules marked by EB3-mCherry. In some cases, multiple Kinesin-1 motility events occurred on a microtubule track that appeared to lie directly adjacent to an EB3-marked dynamic microtubule (boxed region in Figure 2A, kymographs in Figure 2B, 2C). Kinesin-1 motility events overlapped with only 2.5%±1.1% of the EB3-mCherry-marked microtubules (Table 1). These results indicate that the preferential motility of Kinesin-1 motors does not occur on dynamic microtubules.

An avoidance of dynamic microtubules is likely to be advantageous to the motor since the speed of Kinesin-1 is greater than that of microtubule polymerization. In the representative cell shown in Figure 2, KHC(1-560)-3xmCit motors moved at an average speed of 0.73±0.16 µm/sec (Figure 2D, red traces) whereas EB3-labeled microtubules grew at an average rate of 0.08±0.03 µm/sec (Figure 2D, black traces). Thus, Kinesin-1 motors that move along dynamic microtubules could rapidly run off the end of the track.

### Kinesin-1 Motors Move Preferentially on Stable Microtubules Marked by PTMs

**Figure 3:**
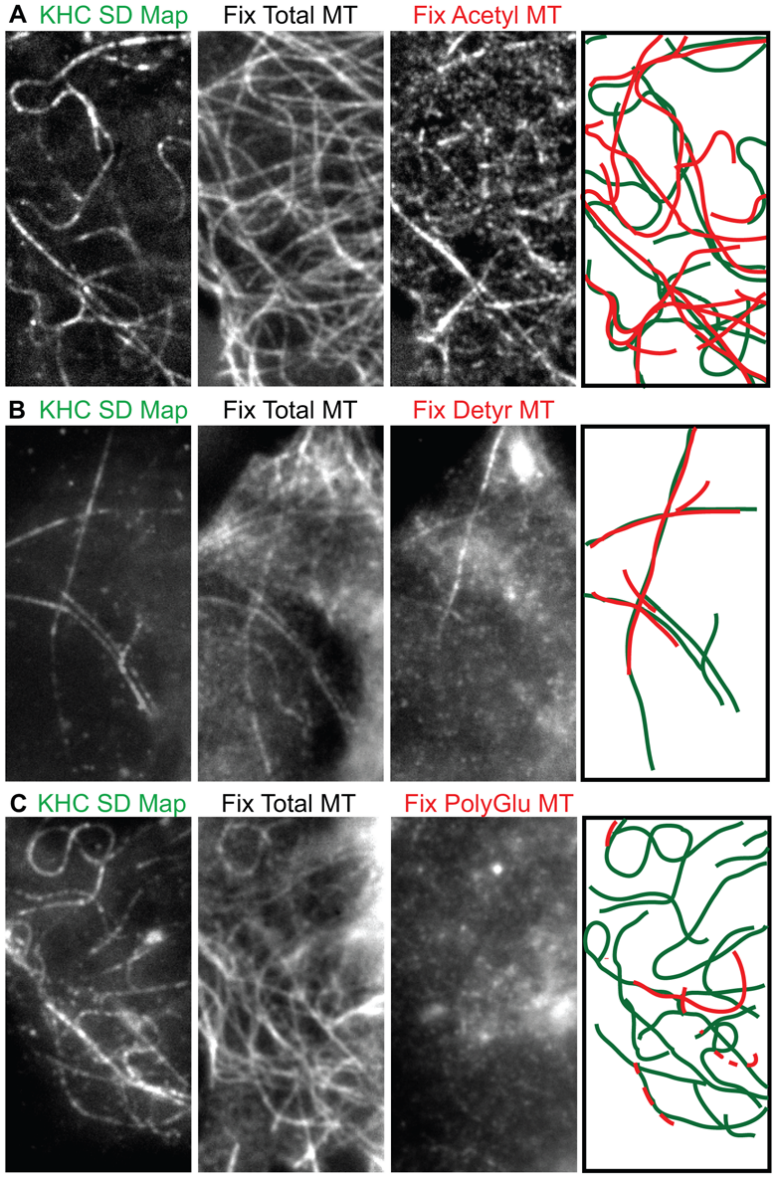
Preferential motility of Kinesin-1 occurs along stable microtubules marked by acetylation and detyrosination of α-tubulin. Single KHC(1-560)-3xmCit motors in live COS cells were imaged by TIRF microscopy. The cells were fixed and stained for retrospective immunofluorescence. SD Maps of the KHC(1-560)-3xmCit motility events were created from the time series (far left images) and compared to the fixed images of (A) total tubulin (middle panel) and acetylated tubulin (right panel), (B) total tubulin (middle panel) and detyrosinated tubulin (right panel), or (C) total tubulin (middle panel) and polyglutamylated tubulins (right panel). Scale bar, 4 µm. The far right panels indicate schematic representations of the overlap between the Kinesin-1 motility events in the SD Map (green lines) and the modified microtubules (red lines).

We then tested whether Kinesin-1 motors move preferentially on stable microtubule tracks. To do this, we performed retrospective immunofluorescence staining using antibodies that recognize the PTMs that mark stable microtubules. Cells expressing KHC(1-560)-3xmCit were imaged in the TIRF microscope, fixed, stained with antibodies to acetylated α-tubulin and total tubulin, and the previously imaged cells were again viewed on the TIRF microscope. The pattern of KHC(1-560)-3xmCit motility events in the resulting SD Map was similar to the pattern of acetylated microtubules (Figure 3A, representative of 11 cells in six experiments). Kinesin-1 motility events colocalized with 90.3%±5.5% of microtubules marked by acetylated tubulin (Table 1). This suggests that Kinesin-1 moves preferentially along stable microtubules marked by acetylation of α-tubulin.

We then used retrospective immunofluorescence to test whether Kinesin-1 motility events correlate with the presence of other PTMs that mark stable microtubules. The pattern of KHC(1-560)-3xmCit motility events in the SD Map was similar to that of the microtubule tracks marked by detyrosination (Figure 3B, representative of six cells in five experiments), a modification that appears to mark the same microtubule tracks as acetylation (see [23] and Figure S3). Kinesin-1 motility events did not colocalize with microtubules marked by polyglutamylation (Figure 3C, representative of eight cells in three experiments), most likely due to the low levels of glutamylation on cytoplasmic microtubules in these (Figure S4) and other non-neuronal cells [24]. We conclude that Kinesin-1 motors move preferentially along microtubules marked by acetylation and detyrosination.

### The Kinesin-2 Motor KIF17 Is a Non-Selective Motor

**Figure 4:**
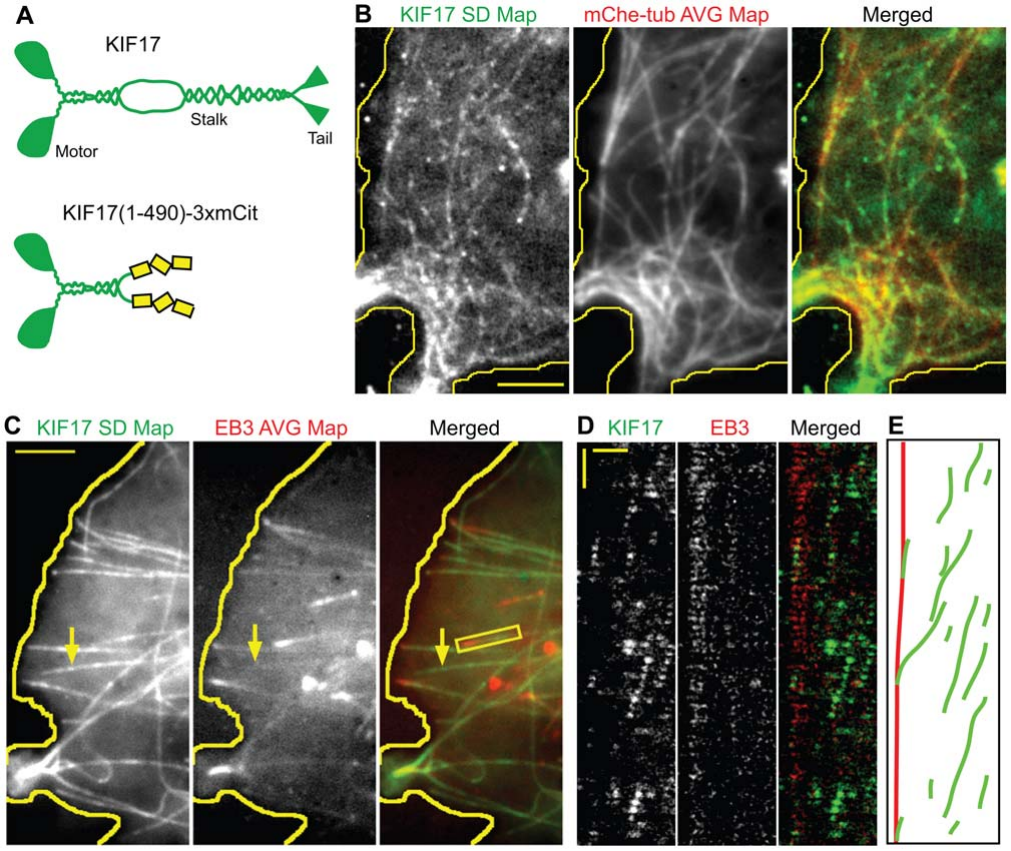
The Kinesin-2 motor KIF17 is a non-selective motor in COS cells. (A) Schematic illustration of the domain structure of full length KIF17 (top) and the truncated, constitutively active version KIF17(1-490)-3xmCit (bottom). (B) Two-color TIRF imaging of KIF17(1-490)-3xmCit motors and mCherry-tubulin in live COS cells. An SD Map of the KIF17(1-490)-3xmCit motility events was created from the time series and compared to the average map of mCherry-tubulin fluorescence. Scale bar, 4 µm. (C) Two-color TIRF imaging of KIF17(1-490)-3xmCit motors and EB3-mCherry in live COS cells. An SD Map of the KHC(1-560)-3xmCit motility events was created from the time series and compared to the average map of EB3-mCherry fluorescence. Arrow, microtubule utilized by KIF17(1-490)-3xmCit but not EB3-mCherry. Yellow line, edge of cell. Scale bar, 4 µm. (D) Kymograph of the boxed region in (C) showing multiple KIF17(1-490)-3xmCit motility events along a single EB3-marked microtubule. Horizontal scale bar, 2 µm. Vertical scale bar, 0.5 s. (E) Schematic diagram of the KIF17 (green) and EB3 (red) tracks in (D).

Is preferential motility on stable microtubules a general feature of kinesin motors that drive vesicular transport events? To test this, we performed single molecule imaging of 3xmCit-tagged KIF17, a homodimeric member of the Kinesin-2 subfamily. KIF17 has been implicated in the transport of cargoes in dendrites of neuronal cells and in cilia of invertebrates and vertebrates [25]–[27]. Single molecule TIRF imaging of a constitutively active version of KIF17 [KIF17(1-490)-3xmCit, Figure 4A] showed that these motors moved with an average speed of 1.31±0.05 µm/sec and average run length of 0.56±0.22 µm in live COS cells (Table 1, *n* = 233 events), consistent with the motile properties of the *C. elegans* homologue OSM-3 [28],[29].

To test whether KIF17 motors move preferentially on a subset of microtubules, we performed two-color TIRF imaging of COS cells co-expressing mCherry-tubulin and KIF17(1-490)-3xmCit (Video S4). A comparison of the SD Map of KIF17(1-490)-3xmCit motility events with the average mCherry-tubulin fluorescence (Figure 4B) demonstrates that KIF17 motility events occurred on nearly all available microtubule tracks (Table 1). In addition, retrospective immunofluorescence imaging indicated that KIF17 motility occurred on both acetylated and non-acetylated microtubules (Table 1 and Figure S5). Together these results indicate that KIF17 is a non-selective motor as it does not show preferential motility when presented with a heterogeneous population of microtubules in COS cells.

Since motility on dynamic microtubules would appear to be disadvantageous to plus end-directed motors, we performed two-color TIRF imaging of COS cells co-expressing KIF17(1-490)-3xmCit and EB3-mCherry to directly test whether KIF17 motors move on dynamic microtubules. A comparison of the SD Map of KIF17 motility events to the average EB3-mCherry fluorescence demonstrates that KIF17 moved on dynamic microtubules (Figure 4C and Table 1). Multiple individual KIF17(1-490)-3xmCit motors could be observed moving on the same EB3-marked microtubule (Figure 4D, 4E). Surprisingly, KIF17 motors that reached the plus end of the microtubule did not dissociate immediately, but rather lingered at the growing plus end (Figure 4E). Whether this ability of KIF17 to track the plus ends of growing microtubules is associated with its cellular functions is presently unknown.

### The Kinesin-3 Motor KIF1A Is a Non-selective Motor

**Figure 5:**
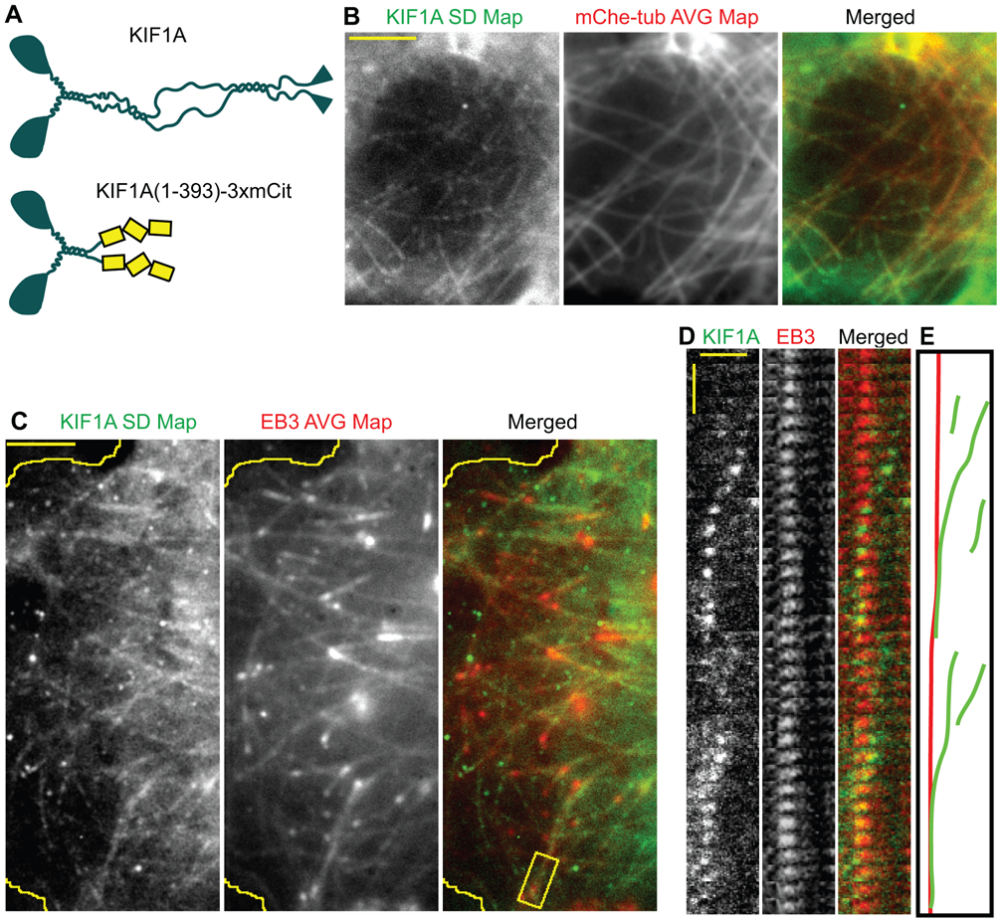
The Kinesin-3 motor KIF1A is a non-selective motor in COS cells. (A) Schematic illustration of the domain structure of full length KIF1A (top) and the truncated, constitutively active version KIF1A(1-393)-3xmCit (bottom). (B) Two-color TIRF imaging of KIF1A(1-393)-3xmCit motors and mCherry-tubulin in live COS cells. An SD Map of the KIF1A(1-393)-3xmCit motility events was created from the time series and compared to the average map of mCherry-tubulin fluorescence. Scale bar, 4 µm. (C) Two-color TIRF imaging of KIF1A(1-393)-3xmCit motors and EB3-mCherry in live COS cells. An SD Map of the KHC(1-560)-3xmCIt motility events was created from the time series and compared to the average map of EB3-mCherry fluorescence. Yellow line, edge of cell. Scale bar, 4 µm. (D) Kymograph of the boxed region in (C) showing multiple KIF1A(1-393)-3xmCit motility events along a single EB3-marked microtubule. Horizontal scale bar, 2 µm. Vertical scale bar, 0.3 s. (E) Schematic diagram of the KIF1A (green) and EB3 (red) tracks in (D).

We then tested whether a member of the Kinesin-3 family, KIF1A, moves preferentially on a subset of microtubules. KIF1A has been implicated in the axonal transport of synaptic vesicle precursors [15]. Constitutively active dimeric KIF1A(1-393)-3xmCit motors (Figure 5A and [30]) imaged by single molecule TIRF microscopy moved with an average speed of 1.82±0.04 µm/sec and average run length of 0.55±0.19 µm in live COS cells (Table 1, *n* = 305 events), consistent with the measured properties of this motor in vitro and its cargoes in vivo [15],[30].

To analyze KIF1A motility on microtubules in vivo, two-color TIRF imaging of COS cells co-expressing mCherry-tubulin and KIF1A(1-393)-3xmCit was performed (Video S5). A comparison of the SD Map of KIF1A(1-393)-3xmCit motility to the average mCherry-tubulin fluorescence indicates that KIF1A motility events were observed on all of the available microtubules (Figure 5B and Table 1) indicating that KIF1A is also a non-selective motor. Consistent with this, KIF1A motility events were observed on both acetylated and non-acetylated microtubules (Table 1 and Figure S5). That KIF1A motors utilize dynamic microtubules for motility was demonstrated by two-color TIRF imaging of COS cells co-expressing KIF1A(1-393)-3xmCit and EB3-mCherry (Figure 5C). Multiple KIF1A motility events were observed to occur on individual dynamic microtubules labeled at their plus ends with EB3-mCherry (Figure 5 D, 5E and Table 1). KIF1A(1-393)-3xmCit motors that overtook the plus end of the microtubules did not fall off but, surprisingly, remained at the plus ends of growing microtubules for significant periods of time (Figure 5E). Thus, the Kinesin-3 motor KIF1A does not select specific microtubule tracks for motility in COS cells and, when using dynamic microtubules, remains localized to the growing plus ends.

### Kinesin-1-Driven Transport Events Occur Preferentially on Stable Microtubules

**Figure 6:**
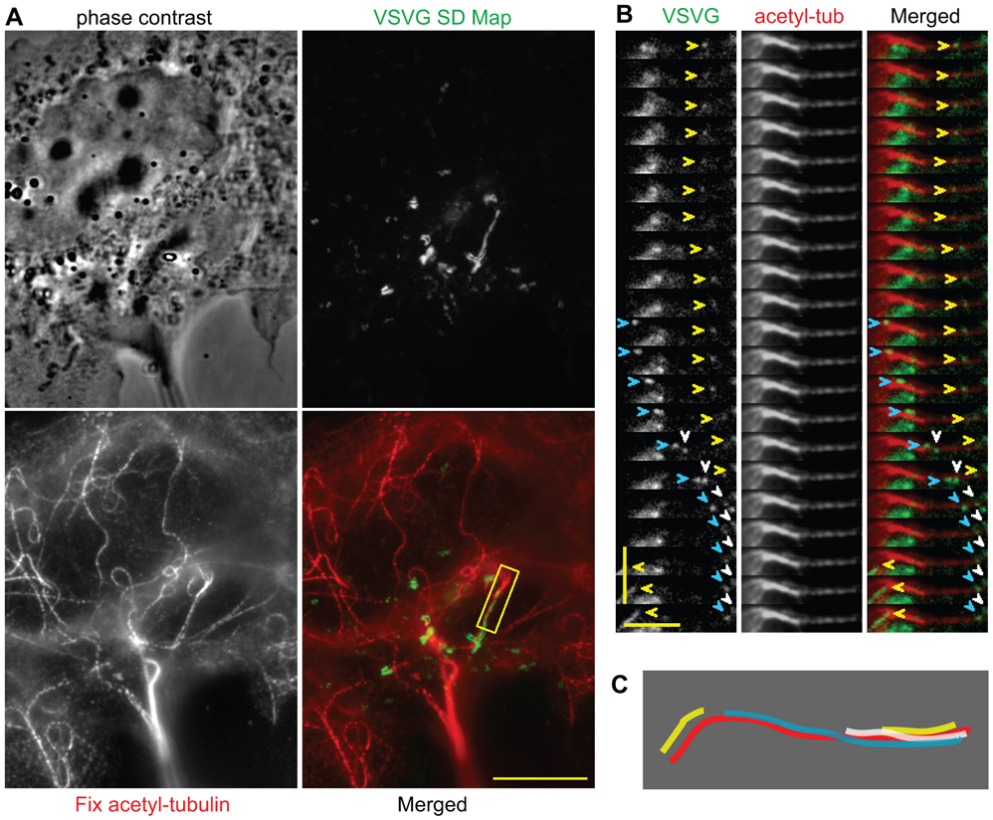
Transport of VSVG-GFP-containing vesicles by Kinesin-1 occurs preferentially along acetylated microtubules. (A) After live cell imaging of Golgi-to-plasma membrane transport of VSVG-GFP, the cells were fixed and stained for retrospective immunofluorescence. An SD Map of the VSVG-GFP vesicle tracks was compared to the image of acetylated microtubules in the same cell. Scale bar, 10 µm. (B) Kymograph of the yellow boxed region in (A). Arrowheads show four VSVG-GFP-containing vesicles being transported along an acetylated microtubule. Horizontal scale bar, 4 µm. Vertical scale bar, 2 s. (C) Schematic diagram summarizing VSVG-GFP-containing vesicles (yellow, white, and cyan traces) undergoing transport on acetylated microtubule (red trace) in (B).

How does the preferential motility of single Kinesin-1 motors on stable microtubules relate to Kinesin-1-driven transport events inside cells? To test whether stable microtubules provide preferred tracks for motility of vesicular cargoes transported by Kinesin-1, we tracked the Golgi-to-plasma membrane transport of a variant of the vesicular stomatitis virus G protein that can be restricted to Golgi-derived vesicles using a temperature shift protocol (VSVG-GFP [19],[31],[32]). After imaging (Video S6), we performed retrospective immunofluorescence with antibodies to total and acetylated tubulin. A comparison of the SD Map of the motility events to the acetylated microtubules in the same cell (Figure 6A, representative of 12 cells in four experiments) demonstrates that 68.0%±15.2% of VSVG-GFP-positive vesicles moved along microtubules marked by acetylated α-tubulin. Multiple VSVG-GFP-marked vesicles were observed to move independently along the same acetylated microtubule (Figure 6B, 6C). These results indicate that Golgi-derived vesicles moved by Kinesin-1 are transported preferentially along microtubules marked by acetylation.

### KIF17-Driven Transport Events Are Not Restricted to Stable Microtubules

We then tested whether cargoes transported by “non-selective” motors, such as KIF1A or KIF17, reflect the properties of these motors. KIF1A transports presynaptic vesicles in neuronal cells, but a cargo for this motor has not been described in fibroblasts. For KIF17, we found that the steady-state cell surface levels of the voltage-gated potassium (Kv) channel Kv1.5 in HL-1 atrial myocytes was decreased by expression of a dominant negative (DN) version of KIF17 but not Kinesin-1 (Figure S6). This was surprising as KIF17 has so far only been described as a dendritic or ciliary motor [15],[33]. Western blot analysis shows that the KIF17 protein is expressed in mouse brain and heart tissues as well as HL-1 myocytes (Figure S7).

**Figure 7:**
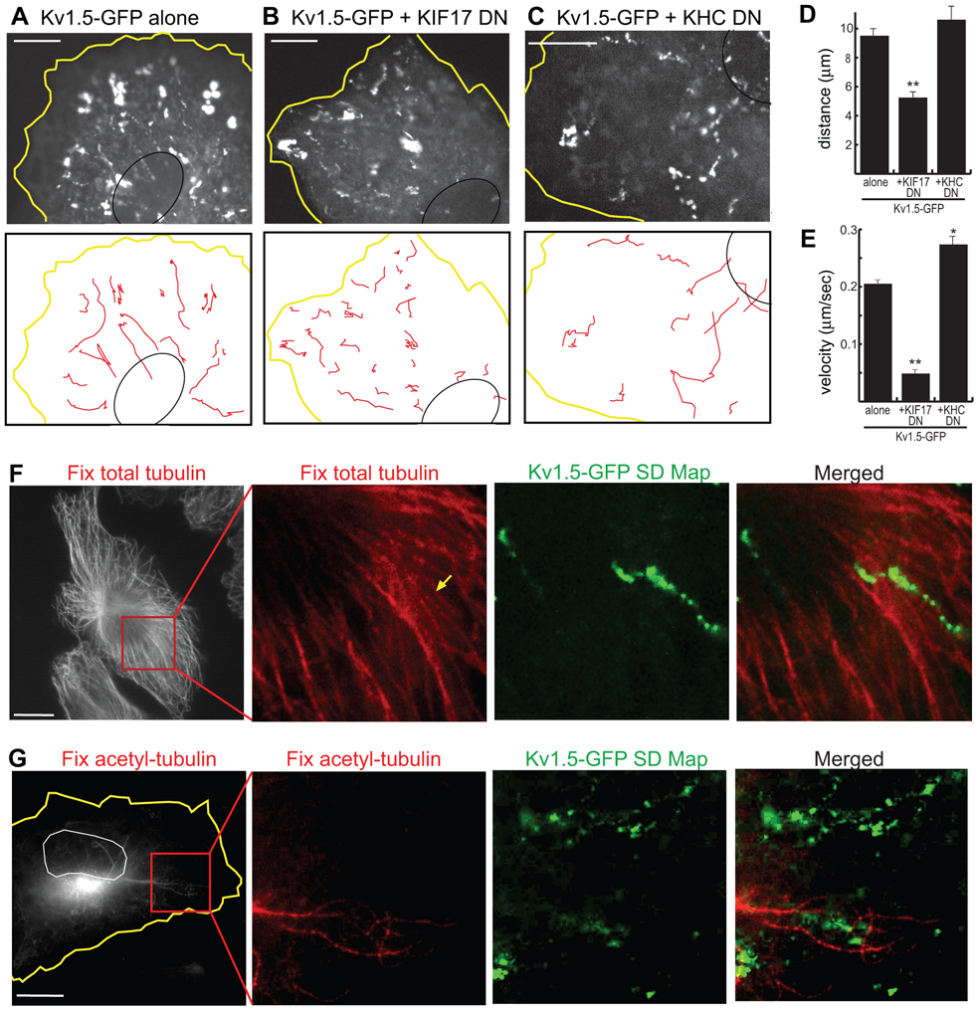
Transport of KV1.5-containing vesicles by KIF17 is not selective for acetylated microtubules. (A–E) Coexpression of a DN KIF17 construct inhibits Kv1.5-GFP channel movement in HL-1 cells. HL-1 cells expressing (A) Kv1.5-GFP, (B) Kv1.5-GFP+mCherry-KIF17-DN, or (C) Kv1.5-GFP+mCherry-KHC-DN were imaged by live cell microscopy. SD Maps (top panels) and schematic drawings (red lines in bottom panels) were generated to visualize Kv1.5-GFP vesicle tracks over time. Yellow line, edge of cell. Black line, nucleus. The average (D) distance traveled and (E) net velocity (includes pauses) of Kv1.5-GFP vesicles under each condition was calculated. Data are mean±SE. Kv1.5-GFP, *n* = 13 cells; Kv1.5-GFP+KIF17-DN, *n* = 17 cells; Kv1.5-GFP+KHC-DN, *n* = 13 cells. *p<0.05, **p<0.001 as compared to Kv1.5-GFP alone. (F,G) Retrospective immunofluorescence of HL-1 cells expressing Kv1.5-GFP. After live cell imaging, an SD Map of the Kv1.5 vesicle tracks was made and compared to the fixed cell images of (F) total microtubules or (G) acetylated microtubules in the same cells. Arrow in (F) indicates microtubule that Kv1.5 vesicle moved on. Yellow line in (G), edge of cell. White line in (G), nucleus. All scale bars, 10 µm.

KIF17 participation in the motility of Kv1.5-GFP labeled vesicles was examined in live cells by coexpressing Kv1.5-GFP with DN versions of KIF17 or Kinesin-1 (KHC) in HL-1 cells (Figure 7A–7C). To synchronize the Kv1.5-GFP vesicle population spatially and temporally, we used a temperature shift protocol to first restrict Kv1.5-GFP to the trans-Golgi by a 19°C incubation and then initiate post-Golgi transport by incubation at 37°C [34]. Golgi-derived Kv1.5-labeled vesicles were observed to move in a linear fashion interspersed with pauses. Expression of DN KIF17, but not DN Kinesin-1, resulted in a significant decrease in Kv1.5 vesicle motility, both in the average distance traveled and average net velocity of the vesicles (Figure 7D, 7E). These results indicate that KIF17 contributes to the microtubule-based transport of Golgi-derived Kv1.5 vesicles in HL-1 myocytes.

To examine whether KIF17-driven motility of Kv1.5 channels occurs along a subset of microtubules, retrospective immunofluorescence imaging was applied to HL-1 myocytes expressing Kv1.5-GFP. After live cell imaging (Video S7), the cells were fixed and stained with antibodies for total or acetylated tubulin. A comparison of the SD Map of Kv1.5-GFP vesicle motility to the image of the total microtubule population demonstrates that Kv1.5 vesicles move along microtubule tracks (Figure 7F, representative of 20 cells in six experiments) but only 13.9%±12.7% of the motility events occurred on microtubules marked by acetylated α-tubulin (Figure 7G, *n* = 3 cells in three experiments). Thus, the motility of Kv1.5 vesicles, like that of the KIF17 motor, does not occur preferentially on stable microtubules marked by acetylation.

## Discussion

That eukaryotic cells contain heterogeneous microtubule populations is widely appreciated. Yet how microtubule diversity is translated into specific microtubule functions is not clear. We show that visualization of single molecules under native physiological conditions reveals new information about how cytoskeletal components interact with each other. Specifically, we show that kinesin motors can translate microtubule diversity into a functional segregation of secretory cargoes.

### Kinesin Motors Differ in their Ability to Select Microtubule Tracks

Our results demonstrate a new property that distinguishes kinesin families—the ability to respond to microtubule heterogeneity in cells. We show that individual Kinesin-1 motors undergo preferential motility along stable microtubules marked by PTMs whereas individual Kinesin-2 (KIF17) and Kinesin-3 (KIF1A) motors are not selective as they undergo motility on both dynamic and stable microtubules.

Transport along stable microtubules would prevent the undesirable situation where a dynamic microtubule track “disappears” under Kinesin-1 and its associated cargoes. Why then do Kinesin-2 and Kinesin-3 motors not avoid dynamic microtubules? One possibility suggested by our live cell imaging of single KIF1A and KIF17 motors (Figures 4 and 5) is that, upon overtaking the plus end of the microtubule, these motors do not dissociate but rather remain at the plus ends of growing microtubules. This may be an important requirement for motors whose cargoes function at the interface of microtubule plus ends and the cell cortex. Interestingly, computer simulations and cell staining have suggested that Kinesin-6 motors may remain attached rather than fall off upon reaching the tips of dynamic microtubules [35],[36]. A second possibility is that these motors and/or their cargoes can prevent depolymerization of the microtubule track. For example, in yeast, transport of +TIP proteins can prevent microtubules from depolymerizing under minus end-directed kinesin motors [37]–[40].

Differences have been reported between kinesin motors in their transport direction in neuronal cells. Kinesin-1 motors accumulate at the tips of axons whereas Kinesin-2 and Kinesin-3 motors accumulate in both axonal and dendritic compartments [18],[19]. Recent work suggests that track selectivity is likely related to the ability to undergo polarized transport [41]. In this work, substitution of residues in the Kinesin-1 motor domain with the corresponding residues of the KIF1A motor domain resulted in a motor that could no longer distinguish between tyrosinated and detyrosinated microtubules in vitro and could not undergo polarized transport to axons in vivo [41]. Thus, a selective motor was converted into a non-selective motor by mutation of the microtubule-binding surface. Together with our work on imaging single motors, these data support the hypothesis that track selection is an inherent property of the motor-microtubule interaction.

### Microtubule-Based Cues for Track Selection by Kinesin-1

What biochemical cues enable Kinesin-1 motors to distinguish microtubule populations? Our results show that Kinesin-1 selects stable microtubules marked by detyrosination and acetylation for preferential motility. One possibility is that it is the PTMs themselves that influence Kinesin-1. This possibility has gained support from recent work in vitro and in vivo [42]–[46]. However, the recognition of PTMs by the Kinesin-1 motor is likely to be complex as mutation of motor surface abolished the ability of Kinesin-1 to recognize detyrosinated but not acetylated microtubules [41]. While glutamylation could play an important role in guiding motor-based transport in epithelial and neuronal cells [44],[47]–[49], it is not likely to play a critical role in fibroblasts. Recent work has indicated that the situation may be different for fungal motors as Kinesin-3 motors, but not Kinesin-1 motors, show track selectivity [50]. Thus, how different PTMs create a tubulin code that can guide motor protein transport events is an important area for future studies.

The correlation of Kinesin-1 motility with specific PTMs does not rule out the possibility that other microtubule-based mechanisms influence this or other motors. Structural changes that occur in the microtubule lattice after polymerization and/or stabilization may influence kinesin motors. Also, MAPs that stabilize microtubules have been shown to negatively influence kinesin-based transport events in cells [51]. Thus, it may be that the modifications that occur along stable microtubules serve to decrease binding of MAPs to microtubules and thus clear the way for motor-based transport [48].

### Segregation of Transport Events between Dynamic and Stable Microtubules

Our work provides the first demonstration that transport events can be segregated between stable and dynamic microtubules via kinesin motors that select these subpopulations of microtubule tracks. Stable microtubules are critical for morphogenesis during diverse biological events such as cytokinesis, cell motility, and neuronal polarity [10],[13],[52],[53]. The preferential motility of Kinesin-1 along stable microtubules may serve to direct Kinesin-1 transport during morphogenesis and maintenance of polarity in neuronal and epithelial cells [18],[19],[41],[44],[54],[55]. A transport module comprised of cargo/Kinesin-1/microtubule subsets is likely involved in other polarized trafficking events such as the delivery of mRNA complexes to the vegetal pole in *Xenopus* oocytes [56].

Dynamic microtubules are important for microtubule search and capture events in mitotic cells as well as in interphase cells during cell polarity and motility [1],[7],[57],[58]. Our results imply that dynamic microtubules can serve as tracks for kinesin-based transport of cargoes that likely function at the microtubule-cortex interface. Indeed, a transport module that comprises cargo/Kinesin-2,3/dynamic microtubule components may be important in trafficking of connexins, cadherins, +TIPs, and channels to cell-cell junctions [59]–[62]. This transport module may also influence retrograde trafficking events such as cytoplasmic dynein-driven movement of endoplasmic reticulum (ER)-derived vesicles to the central Golgi complex [63].

This work is the first to analyze the segregation of kinesin motors and their cargoes to distinct microtubules populations and subcellular destinations. Recent work using TIRF microscopy of detergent-extracted cells has indicated that unconventional myosins also differ in their ability to select actin filament tracks [64]. Thus, these types of experiments provide a starting point for exploring the ability of motor proteins to respond to structural and/or biochemical changes in cytoskeletal filaments inside cells as well as the relationship between track selection and cellular function.

## Materials and Methods

### Cell Culture and Reagents

COS and HL-1 cells were cultured and transfected as described [17],[65]. The following antibodies were purchased: total β-tubulin (E7, Developmental Studies Hybridoma Bank, Univ. Iowa), acetylated α-tubulin (Sigma B-11-61), detyrosinated α-tubulin (Chemicon), GFP (Invitrogen), and fluorescently marked secondary antibodies (Jackson ImmunoResearch). A monoclonal antibody to polyglutamylated tubulin (GT335) was a gift from C Janke (CNRS, France). A polyclonal antibody that recognizes acetylated α-tubulin was generated against amino acids 29–52.

### Plasmids

Constitutively active versions of kinesin motors were generated by PCR cloning of the relevant sequences (aa 1-490 of human KIF17, aa 1-393 of rat KIF1A, and aa 1-560 of rat KIF5C) into the 3xmCit-N1 vector [17]. DN versions of KHC (aa 566-955) and KIF17 (aa 488-846) were cloned into mCherry-C1. All plasmids were verified by DNA sequencing. Plasmids encoding VSVG-GFP and EB3-mCherry were gifts from A Akhmanova and N Galjart (U Rotterdam). Kv1.5-GFP has been described [65].

### TIRF Microscopy

Objective-based TIRF microscopy was carried out as described [17]. Briefly, transfected COS cells on a glass-bottomed 35 mm dish (MatTek) were carefully rinsed with Ringers buffer (10 mM HEPES/KOH, 155 mM NaCl, 5 mM KCl, 2 mM CaCl_2_, 1 mM MgCl_2_, 2 mM NaH_2_PO_4_, 10 mM glucose, pH 7.2) and imaged on a custom-modified Zeiss Axiovert 135TV microscope equipped with a 1.45 NA a-Plan Fluor objective, 2.5× optovar, 505DCXR dichroic and HQ510LP emission filter (Chroma Technology), 488 nm line of a tunable single-mode fiber-coupled Argon Ion Laser (Melles Griot), and a back-illuminated EMCCD camera (Cascade 512B, Roper Scientific). The angle of illumination was adjusted for maximum penetration of the evanescent field into the cell (near-TIRF), enabling an imaging depth of ∼500 nm, which is sufficient to image nearly all the microtubules in the periphery of flat COS cells. For two-color TIRF, a yellow diode pumped solid-state laser (593 nm, CrystaLaser) was combined with the 488 nm laser using a dichroic mirror (Z488RDC). Fluorescence emissions were first passed though a FF495/605 dual-band dichroic mirror (Semrock) and then projected onto separate halves of the CCD camera by a Dualview beam-splitter (Optic Insights) equipped with a T585LP dichroic beam splitter and ET525/50M and HQ610LP emission filters (all Chroma Technology). In general, cells were imaged ∼6 h post-transfection to maintain low expression levels representative of endogenous protein behavior and optimal for single molecule TIRF imaging. Images were captured every 50–100 ms for 30–35 s. All experiments were carried out at room temperature (18–21°C). For measurement of EB3-mCherry microtubules, the lower average microtubule growth rate compared to those reported in other studies (e.g. [66] and references therein) is likely due to this lower temperature.

### Retrospective Immunofluorescence

Immediately after imaging, cells were fixed (3 min) in 3.7% paraformaldehyde (Ted Pella), quenched (10 min) with 50 mM NH_4_Cl, and permeabilized (3 min) with 0.2% Triton X-100. An equal volume of 1.0% glutaraldehyde was then carefully added for an additional 7 min of fixation. After quenching with freshly prepared 1.5 mg/ml NaBH_4_, primary antibodies in 0.2% fish skin gelatin were incubated for 1–2 h at room temperature or overnight at 4°C.

### Wide-Field Microscopy

COS or HL-1 cells in glass-bottom dishes (MatTek) were imaged live on a Nikon TE2000 microscope with a Plan-APO 100×/NA 1.4 objective and Photometrics CS ES2 camera. VSVG(tsO45)-GFP-expressing cells were incubated overnight at 39°C to accumulate protein in the ER. Two h after shifting cells to 33°C for synchronous protein transport through the Golgi complex and to the plasma membrane, images were obtained every 1 s. For Kv1.5-GFP, cells were incubated at 19°C for 3 h to accumulate secretory proteins in the trans-Golgi and then shifted to 37°C and imaged every 5 s. Statistical analysis was done using one-way ANOVA analysis.

### Off-Line Imaging Processing

Videos and images were prepared with ImageJ (NIH) and Photoshop and Illustrator (Adobe). Generation of the SD Maps is described in Figure S1 and [17]. Home-made plug-ins for ImageJ were used for measuring the speed and run length of motors and vesicles. For motors, only diffraction-limited fluorescence spots (5×5 pixels) were selected for analysis that were clearly separated from the neighboring fluorescence and moved in a linear fashion on microtubules tracks identified in the SD Maps. Motile events that did not appear in the SD Map were usually short and/or blurry events that could not be separated from diffusion. For colocalization of motor and microtubule tracks (Table 1), the SD Map of the motility events was overlaid with a static image of the mCherry-tubulin fluorescence. The relative overlap (expressed in %) was calculated as the ratio of microtubules with motility events to total number of microtubules. For colocalization of VSVG-GFP or Kv1.5-GFP vesicles with acetylated tubulin, the SD Map of the vesicle motility was overlaid with the fixed acetylated tubulin image. The overlap (expressed in %) was calculated as the (number of vesicles on microtubules)/(number moving vesicles).

Supplemental methods are described in Text S1.

## Supporting Information

Figure S1 **Generation of an SD Map from an image series.** A time series of images is obtained in the TIRF microscope for an area in the periphery of a COS cell expressing FP-tagged proteins of interest. To highlight the Kinesin-1 motility events during this time series, an SD Map is then calculated from the image series by calculating the statistics of the variation in fluorescence intensity for each pixel location in the raw images. For an image stack containing Z slices of images, the SD of the intensity (I) of each pixel was calculated with ImageJ (ZProjector_StandardDeviation) and then plotted in the form of one image referred to as the SD Map. Yellow line in SD Map, edge of cell. Scale bar, 3 µm.
(2.10 MB TIF)

Figure S2 **Individual microtubules can shift their position during live cell imaging.** (A) Shifts in the positioning of individual microtubules can be observed during live cell TIRF imaging of COS cells expressing mCherry-tubulin. Still images taken at 0 s and 0.5 s are shown. A merge of the two images (right panel: 0 s, green; 0.5 s, red) shows that while most of the microtubules remained stationary during the imaging, individual microtubules (arrows) shifted position. Scale bar, 2 µm. (B) Shifts in the position of individual microtubules can also be seen during live imaging of single kinesin motors. COS cells expressing KHC(1-560)-3xmCit were imaged live by TIRF microscopy. Images were collected every 100 ms. SD Maps were generated from the 20 frames following the time stamp indicated in each panel. Arrowhead, microtubule that shifts in position while serving as a track for kinesin motors. Scale bar, 3 µm.
(4.49 MB TIF)

Figure S3 **Colocalization of acetylated and detyrosinated microtubules.** COS cells were fixed and stained with antibodies to acetylated and detyrosinated tubulins. Scale bars, 5 µm.
(3.86 MB TIF)

Figure S4 **Microtubule polyglutamylation in COS cells.** COS cells were fixed and stained with antibodies to total and polyglutamylated tubulins. Microtubule polyglutamylation is found in the primary cilium (arrowhead, top row) and centrosome (arrow, top row) as well as the mitotic spindle (star, bottom row) and midbody (asterisks, bottom row). Scale bar, 10 µm.
(2.09 MB TIF)

Figure S5 **Kinesin-2 and Kinesin-3 motors are not selective for stable microtubules marked by acetylation of α-tubulin.** COS cells expressing (A) KIF17(1-490)-3xmCit or (B) KIF1A(1-393)-3xmCit motors were imaged live by TIRF microscopy. The cells were fixed and stained for retrospective immunofluorescence with an antibody to acetylated tubulin. SD Maps of the kinesin motility events were created from the time series and compared to the fixed images of acetylated tubulin. Kinesin-2 and Kinesin-3 motility events can be seen to occur on both acetylated and non-acetylated microtubules. Scale bar, 3 µm.
(2.88 MB TIF)

Figure S6 **Expression of a DN KIF17 construct results in decreased steady-state surface levels of Kv1.5 channels.** HL-1 cells were transiently transfected with Kv1.5-GFP plasmid alone or cotranfected with plasmids for mCherry-KIF17-DN or mCherry-KHC-DN. Kv1.5 channels at the cell surface were detected by staining live cells at 4°C with anti-GFP primary and Alexa647 secondary antibodies. The cells were then fixed and imaged. Surface Kv1.5 (Alexa647 fluorescence) was normalized to the total Kv1.5 population (GFP fluorescence) for each condition. The average levels of surface Kv1.5 in the control population was set at 100%. All data are presented as the mean±SE of three experiments (at least 30 cells total each). The presence of KIF17-DN, but not KHC-DN, reduces Kv1.5 levels at the plasma membrane (A) although the total channel levels are comparable across conditions (B). Scale bars, 10 µm. **p<0.05.
(0.47 MB TIF)

Figure S7 **KIF17 and Kinesin-1 proteins are both expressed in the atrium.** Tissues or cells were separated by SDS-PAGE electrophoresis and transferred to nitrocellulose for Western Blot analysis. Lanes: 1, mouse brain lysate; 2, mouse liver lysate; 3, mouse atrium lysate; 4, HL-1 cell lysate. The size (kDa) of the molecular weight markers is shown on the left of each gel. (A) Blots were probed with an anti-KIF17 polyclonal antibody (Abcam). Arrow indicates specific band for KIF17. (B) Blot in (A) was stripped and re-probed with a polyclonal antibody to the kinesin motor domain that recognizes the motor domain of Kinesin-1 (arrow) as well as similar sequences in other kinesin family members. (C) Blot in (A) and (B) was stripped and re-probed with an anti-α-tubulin polyclonal antibody to ensure equal protein loading. Arrow indicates specific band for α-tubulin.
(0.87 MB TIF)

Text S1 **Supplemental methods.** Additional information concerning methods to determine cell surface levels of Kv1.5 and Western Blotting of Kv1.5
(0.05 MB DOC)

Video S1 **Image series taken by single molecule TIRF imaging in the periphery of a COS cell expressing KHC(1-560)-3xmCit.**
(2.44 MB MOV)

Video S2 **Two-color TIRF imaging was carried out in the periphery of a COS cell coexpressing KHC(1-560)-3xmCit and mCherry-tubulin.** The two movies are merged to show motility of KHC (kinesin-1) motors along microtubule tracks.
(1.47 MB MOV)

Video S3 **Two-color TIRF imaging in the periphery of a COS cell coexpressing KHC(1-560)-3xmCit (green) and EB3-mCherry (red).** The movie is an overlay of the two image sequences. KHC(1-560)-3xmCit motors do not move on dynamic microtubules extending back from EB3-mCherry plus ends.
(4.04 MB MOV)

Video S4 **Two-color TIRF imaging was carried out in the periphery of a COS cell coexpressing KIF17(1-490)-3xmCit (green) and mCherry-tubulin (red).** The movie is an overlay of the two image sequences and shows motility of KIF17 (Kinesin-2) motors along microtubule tracks.
(8.22 MB MOV)

Video S5 **Two-color TIRF imaging was carried out in the periphery of a COS cell coexpressing KIF1A(1-393)-3xmCit and mCherry-tubulin.** The movie is an overlay of the two image sequences and shows motility of KIF1A (Kinesin-3) motors along microtubule tracks.
(6.52 MB MOV)

Video S6 **Live cell wide-field imaging of a COS cell expressing VSVG-GFP (Kinesin-1 cargo) 2 h after shift to the permissive temperature.** An image of the acetylated microtubules (red) in the same cell was obtained by fixation and retrospective immunostaining with an antibody to acetylated tubulin. The movie is an overlay of the image sequence of VSVG-GFP vesicles on the image of acetylated microtubules.
(4.58 MB MOV)

Video S7 **Live cell wide-field imaging of an HL-1 atrial myocyte expressing Kv1.5-GFP (Kinesin-2 cargo).** An image of the total microtubules (red) in the same cell was obtained by fixation and retrospective immunostaining with an antibody to total β-tubulin. The movie is an overlay of the image sequence of Kv1.5-GFP vesicles on the image of total microtubules.
(1.01 MB MOV)

## Abbreviations

DN: dominant negative
EB: end binding
FP: fluorescent protein
KHC: Kinesin heavy chain
KLC: Kinesin light chain
MAP: microtubule associated protein
mCit: monomeric Citrine
+TIP: plus end tracking protein
PTM: post-translational modification
SD: standard deviation
TIRF: total internal reflection fluorescence
VSVG: vesicular stomatitis virus G protein
