# Supplementary figures and images for: Single Molecule Imaging Reveals Differences in Microtubule Track Selection Between Kinesin Motors
Source: PLoS Biol. 2009 Oct 13;7(10):e1000216. doi: 10.1371/journal.pbio.1000216 (PMC2749942; doi:10.1371/journal.pbio.1000216)

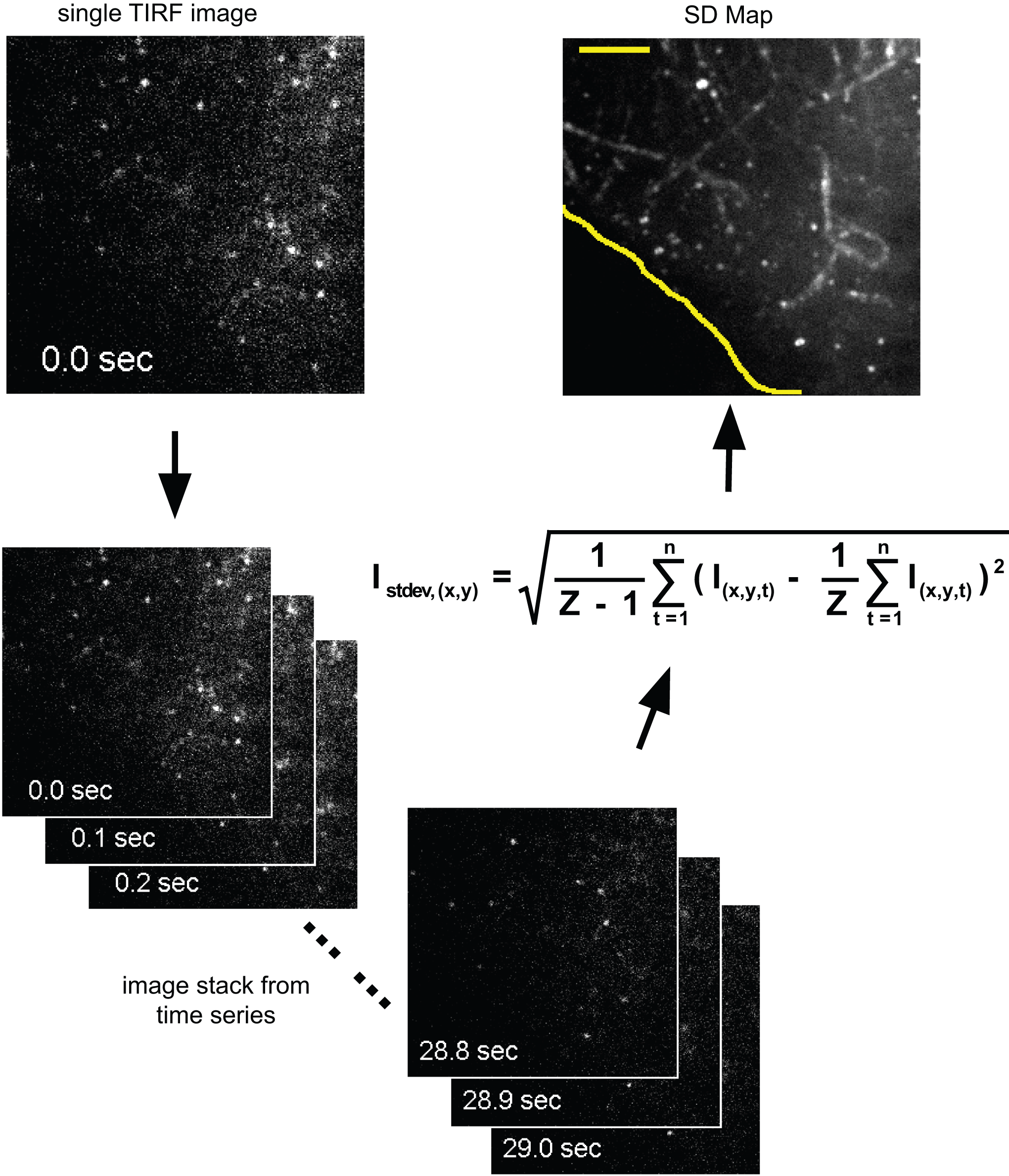

Supplement: Figure S1 — Generation of an SD Map from an image series. A time series of images is obtained in the TIRF microscope for an area in the periphery of a COS cell expressing FP-tagged proteins of interest. To highlight the Kinesin-1 motility events during this time series, an SD Map is then calculated from the image series by calculating the statistics of the variation in fluorescence intensity for each pixel location in the raw images. For an image stack containing Z slices of images, the SD of the intensity (I) of each pixel was calculated with ImageJ (ZProjector_StandardDeviation) and then plotted in the form of one image referred to as the SD Map. Yellow line in SD Map, edge of cell. Scale bar, 3 µm. (2.10 MB TIF) [file pbio.1000216.s001.tif]

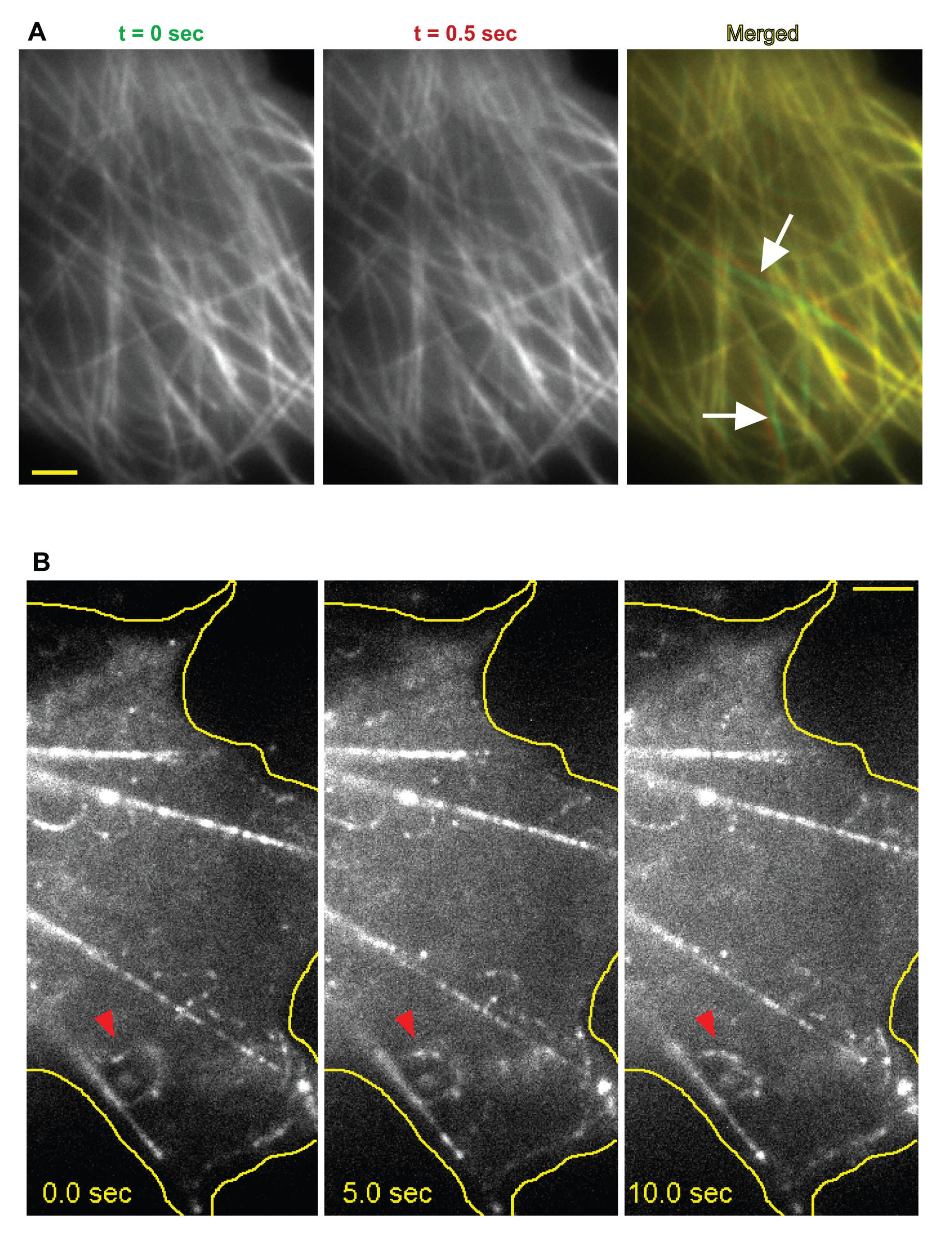

Supplement: Figure S2 — Individual microtubules can shift their position during live cell imaging. (A) Shifts in the positioning of individual microtubules can be observed during live cell TIRF imaging of COS cells expressing mCherry-tubulin. Still images taken at 0 s and 0.5 s are shown. A merge of the two images (right panel: 0 s, green; 0.5 s, red) shows that while most of the microtubules remained stationary during the imaging, individual microtubules (arrows) shifted position. Scale bar, 2 µm. (B) Shifts in the position of individual microtubules can also be seen during live imaging of single kinesin motors. COS cells expressing KHC(1-560)-3xmCit were imaged live by TIRF microscopy. Images were collected every 100 ms. SD Maps were generated from the 20 frames following the time stamp indicated in each panel. Arrowhead, microtubule that shifts in position while serving as a track for kinesin motors. Scale bar, 3 µm. (4.49 MB TIF) [file pbio.1000216.s002.tif]

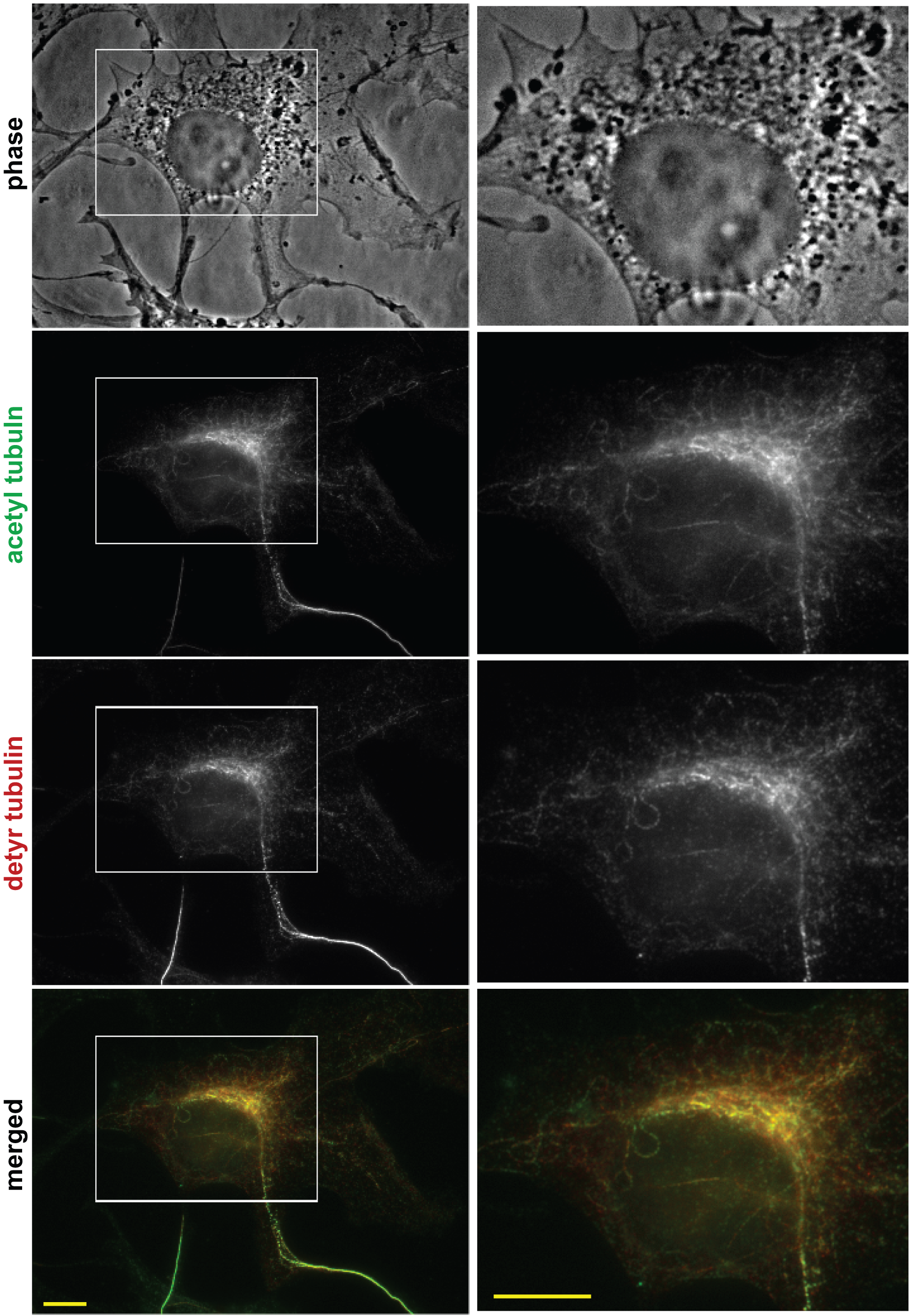

Supplement: Figure S3 — Colocalization of acetylated and detyrosinated microtubules. COS cells were fixed and stained with antibodies to acetylated and detyrosinated tubulins. Scale bars, 5 µm. (3.86 MB TIF) [file pbio.1000216.s003.tif]

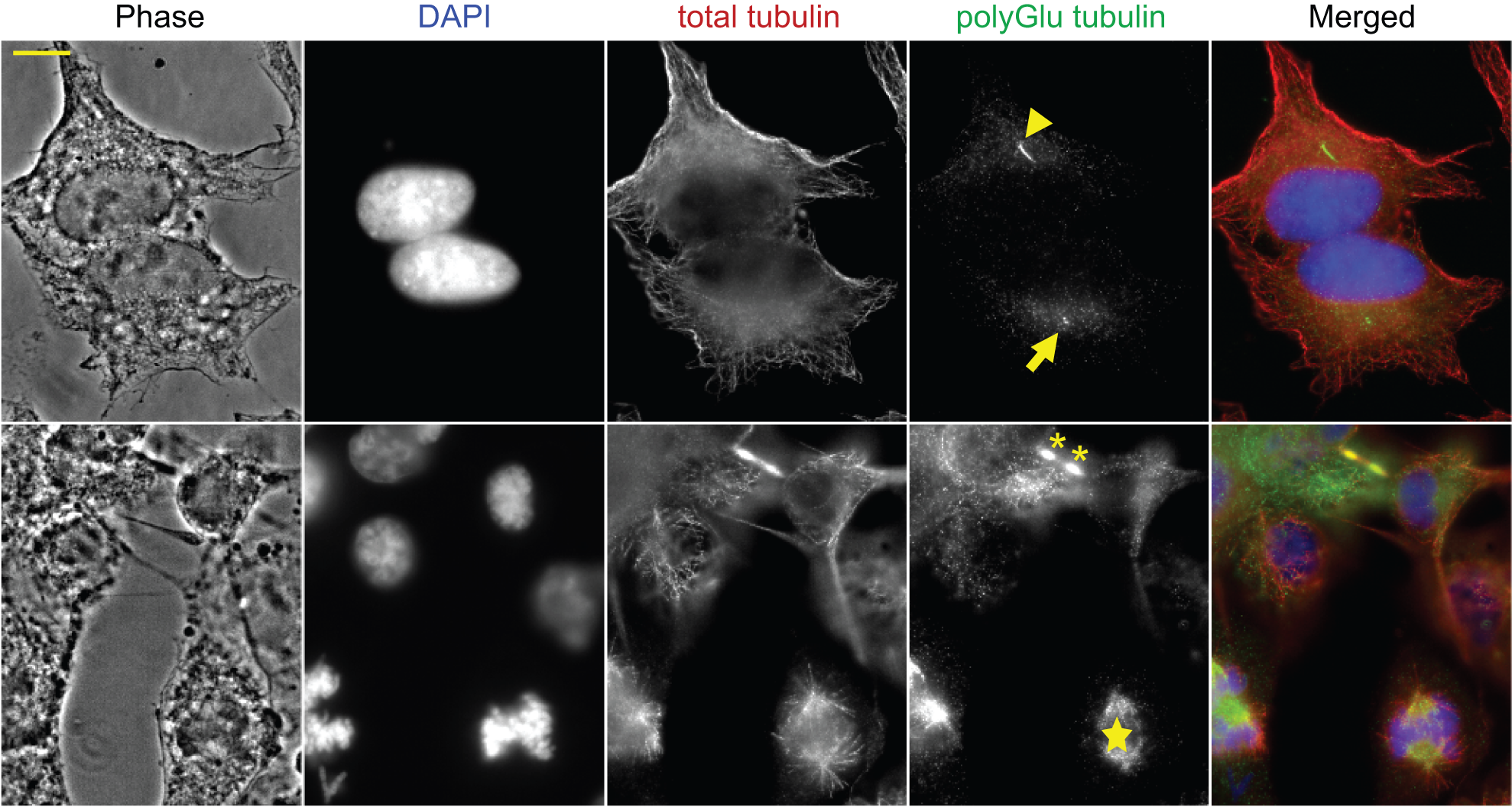

Supplement: Figure S4 — Microtubule polyglutamylation in COS cells. COS cells were fixed and stained with antibodies to total and polyglutamylated tubulins. Microtubule polyglutamylation is found in the primary cilium (arrowhead, top row) and centrosome (arrow, top row) as well as the mitotic spindle (star, bottom row) and midbody (asterisks, bottom row). Scale bar, 10 µm. (2.09 MB TIF) [file pbio.1000216.s004.tif]

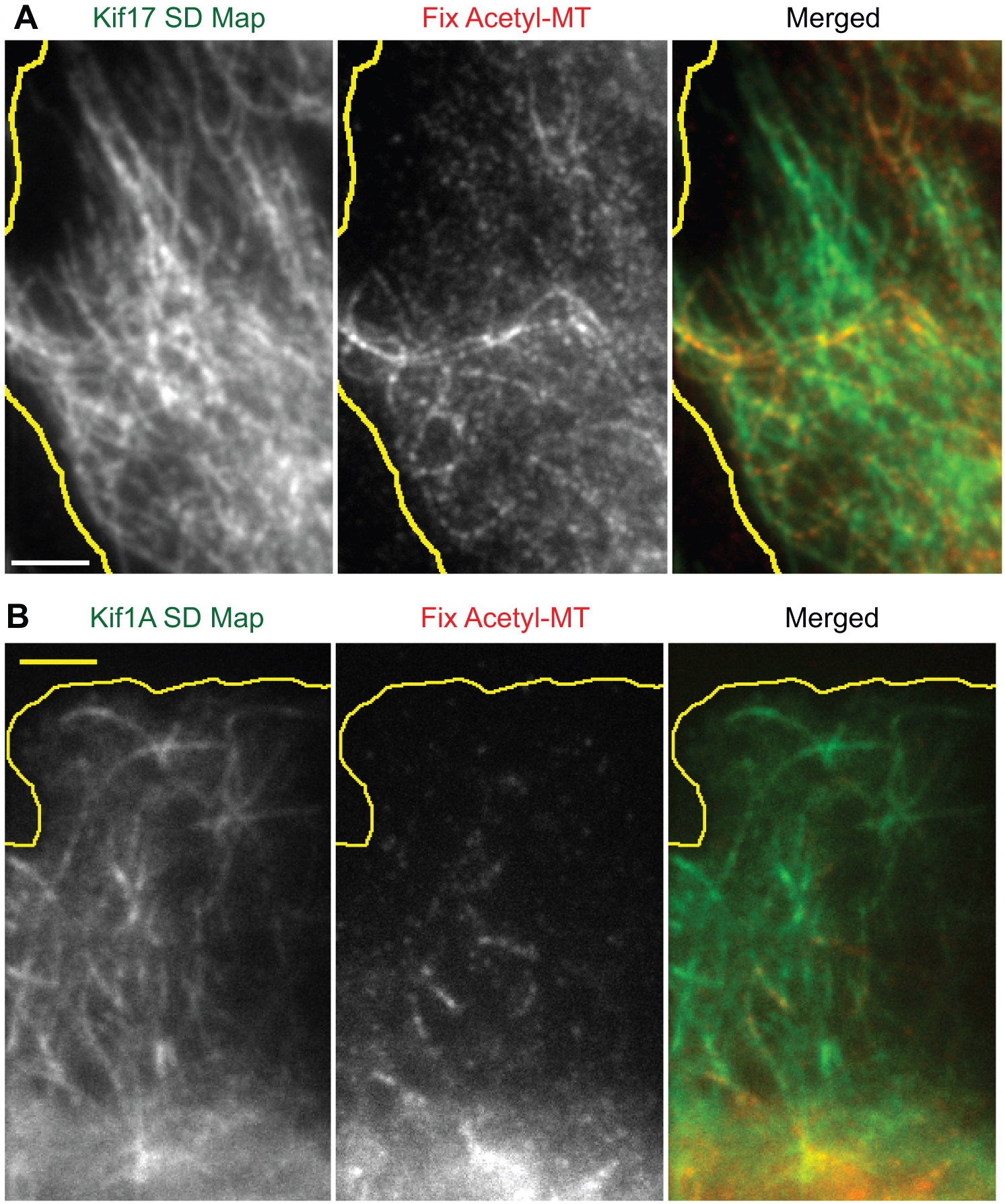

Supplement: Figure S5 — Kinesin-2 and Kinesin-3 motors are not selective for stable microtubules marked by acetylation of α-tubulin. COS cells expressing (A) KIF17(1-490)-3xmCit or (B) KIF1A(1-393)-3xmCit motors were imaged live by TIRF microscopy. The cells were fixed and stained for retrospective immunofluorescence with an antibody to acetylated tubulin. SD Maps of the kinesin motility events were created from the time series and compared to the fixed images of acetylated tubulin. Kinesin-2 and Kinesin-3 motility events can be seen to occur on both acetylated and non-acetylated microtubules. Scale bar, 3 µm. (2.88 MB TIF) [file pbio.1000216.s005.tif]

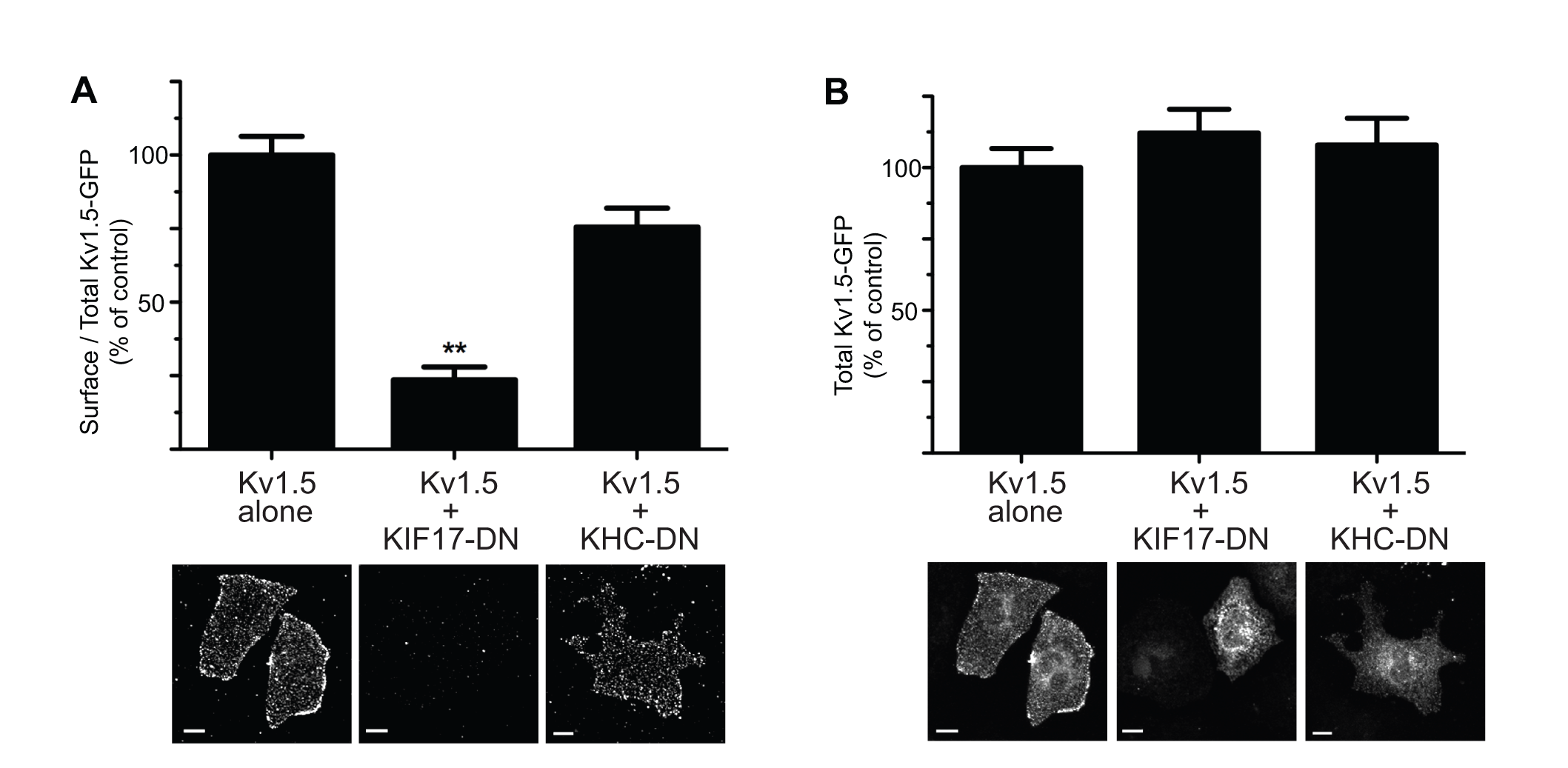

Supplement: Figure S6 — Expression of a DN KIF17 construct results in decreased steady-state surface levels of Kv1.5 channels. HL-1 cells were transiently transfected with Kv1.5-GFP plasmid alone or cotranfected with plasmids for mCherry-KIF17-DN or mCherry-KHC-DN. Kv1.5 channels at the cell surface were detected by staining live cells at 4°C with anti-GFP primary and Alexa647 secondary antibodies. The cells were then fixed and imaged. Surface Kv1.5 (Alexa647 fluorescence) was normalized to the total Kv1.5 population (GFP fluorescence) for each condition. The average levels of surface Kv1.5 in the control population was set at 100%. All data are presented as the mean±SE of three experiments (at least 30 cells total each). The presence of KIF17-DN, but not KHC-DN, reduces Kv1.5 levels at the plasma membrane (A) although the total channel levels are comparable across conditions (B). Scale bars, 10 µm. **p<0.05. (0.47 MB TIF) [file pbio.1000216.s006.tif]

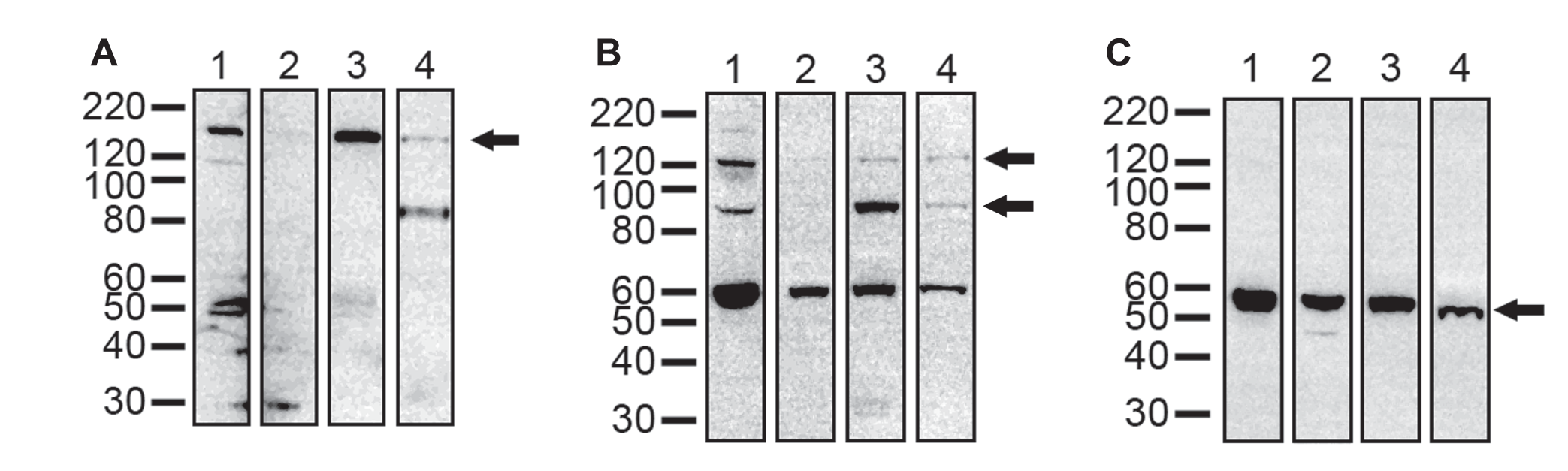

Supplement: Figure S7 — KIF17 and Kinesin-1 proteins are both expressed in the atrium. Tissues or cells were separated by SDS-PAGE electrophoresis and transferred to nitrocellulose for Western Blot analysis. Lanes: 1, mouse brain lysate; 2, mouse liver lysate; 3, mouse atrium lysate; 4, HL-1 cell lysate. The size (kDa) of the molecular weight markers is shown on the left of each gel. (A) Blots were probed with an anti-KIF17 polyclonal antibody (Abcam). Arrow indicates specific band for KIF17. (B) Blot in (A) was stripped and re-probed with a polyclonal antibody to the kinesin motor domain that recognizes the motor domain of Kinesin-1 (arrow) as well as similar sequences in other kinesin family members. (C) Blot in (A) and (B) was stripped and re-probed with an anti-α-tubulin polyclonal antibody to ensure equal protein loading. Arrow indicates specific band for α-tubulin. (0.87 MB TIF) [file pbio.1000216.s007.tif]
