# Supplementary material for: Single Molecule Imaging Reveals Differences in Microtubule Track Selection Between Kinesin Motors
Source: PLoS Biol. 2009 Oct 13;7(10):e1000216. doi: 10.1371/journal.pbio.1000216 (PMC2749942; doi:10.1371/journal.pbio.1000216)
Supplement: Text S1 — Supplemental methods. Additional information concerning methods to determine cell surface levels of Kv1.5 and Western Blotting of Kv1.5 (0.05 MB DOC) [file pbio.1000216.s008.doc]

**Supplemental Methods**

*Steady-state Kv1.5 surface immunocytochemistry.*  48 hr post-transfection, HL-1 cells were washed with PBS and stained with anti-GFP antibodies (Invitrogen, 1:500) for 30’ on ice to label surface proteins. Cells were then washed with PBS, incubated with goat anti-rabbit AlexaFluor647 secondary antibody (1:500) in 2% goat serum for 30 min on ice, washed with PBS, and then fixed with 4% paraformaldehyde and mounted with ProLong Gold anti-fade reagent (Invitrogen). All images were collected on an Olympus FluoView 500 confocal microscope. Z-stacks were compressed and total fluorescence was calculated for total Kv1.5 (GFP) and surface Kv1.5 (AlexaFluor) using NIH ImageJ software. Background fluorescence was determined by measuring the fluorescent signal in neighboring untransfected HL-1 cells for all fluorescent channels tested (GFP and AlexaFluor) and was subtracted from the total fluorescent signal. The ratio of surface Kv1.5 fluorescent signal to total Kv1.5-GFP fluorescence in each cell was then determined.

*Western Blotting*. For mice, tissues were excised and homogenized by Dounce homogenization in ice-cold lysis buffer (50 mM Tris/HCl, 150 mM NaCl, 10 mM EDTA, pH 8.0, 1% Triton X-100) containing protease inhibitors. For all lysates, 50 μg of protein was separated by SDS-PAGE on a NuPAGE Novex 4-12% bis-tris gel (Invitrogen). Proteins were transferred to nitrocellulose and probed with the indicated primary antibody for 1 h at room temperature. Blots were then incubated with secondary antibodies conjugated to horseradish peroxidase and visualized using the Western lightning enhanced chemiluminescent reagent according to the manufacturer's protocol (PerkinElmer Life Sciences). Images were captured using the EpiChemi3 darkroom (UVP, Inc., Upland, CA).
